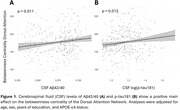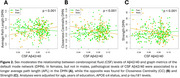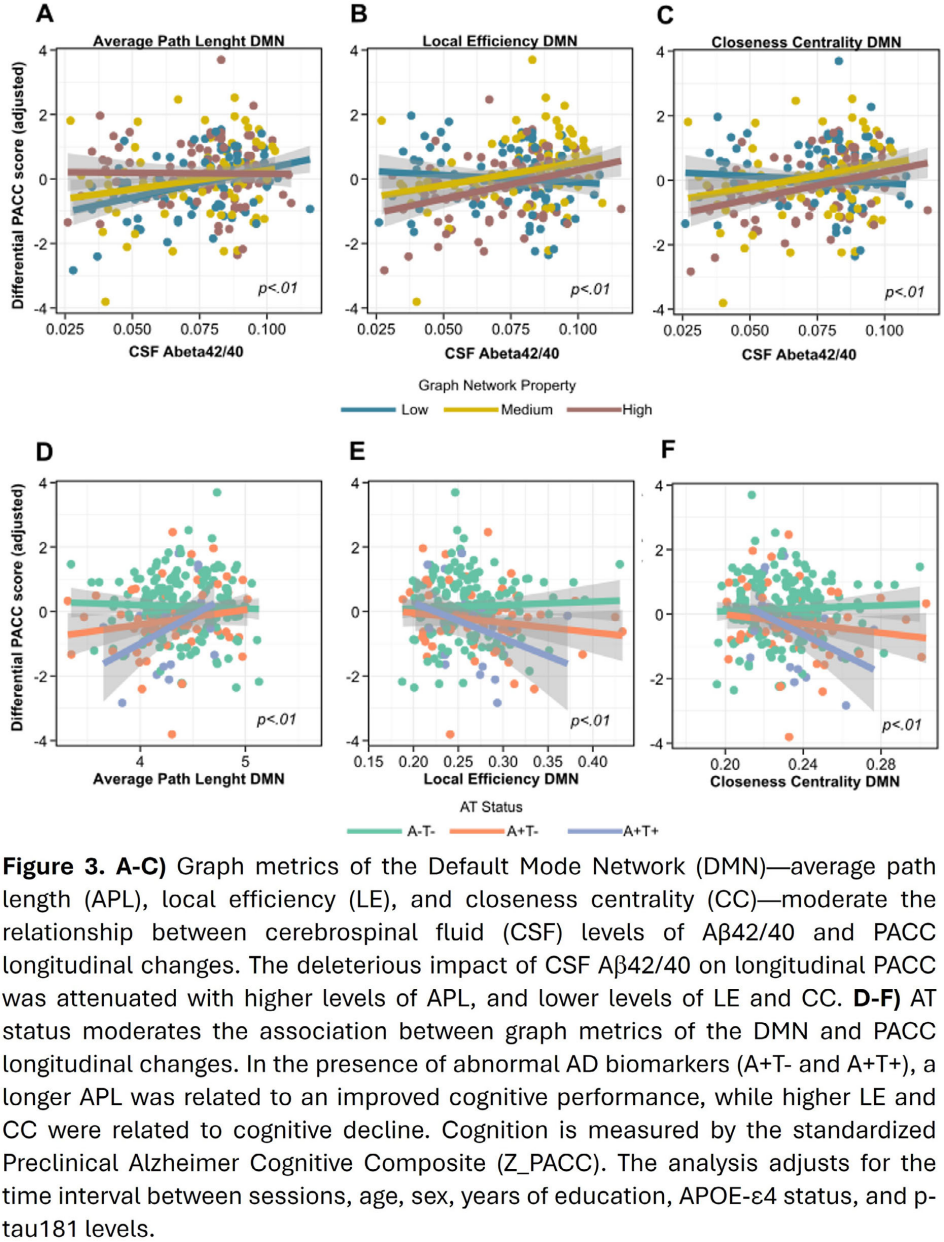# Association of graph network properties with Alzheimer's pathological hallmarks early in the disease continuum

**DOI:** 10.1002/alz70856_099727

**Published:** 2025-12-25

**Authors:** Aldana Lizarraga, Michalis Kassinopoulos, José María González‐de‐Echávarri, Jordi Huguet, Gonzalo Sánchez‐Benavides, Oriol Grau‐Rivera, Marc Suarez‐Calvet, Marta Milà‐Alomà, Kaj Blennow, Henrik Zetterberg, Gwendlyn Kollmorgen, Clara Quijano‐Rubio, Juan Domingo Gispert, Gemma Salvadó, Raffaele Cacciaglia

**Affiliations:** ^1^ Barcelonaβeta Brain Research Center (BBRC), Pasqual Maragall Foundation, Barcelona, Spain; ^2^ Hospital del Mar Research Institute, Barcelona, Spain; ^3^ Centro de Investigación Biomédica en Red de Fragilidad y Envejecimiento Saludable (CIBERFES), Madrid, Spain; ^4^ BarcelonaBeta Brain Research Center (BBRC), Barcelona, Spain; ^5^ Centro de Investigación Biomédica en Red de Fragilidad y Envejecimiento Saludable (CIBERFES), Instituto de Salud Carlos III, Barcelona, Spain; ^6^ Servei de Neurologia, Hospital del Mar, Barcelona, Spain; ^7^ Hospital del Mar Research Institute (IMIM), Barcelona, Spain; ^8^ Department of Veterans Affairs Medical Center, Northern California Institute for Research and Education (NCIRE), San Francisco, CA, USA; ^9^ Clinical Neurochemistry Laboratory, Sahlgrenska University Hospital, Mölndal, Sweden; ^10^ Department of Psychiatry and Neurochemistry, University of Gothenburg, Gothenburg, Sweden; ^11^ Clinical Neurochemistry Laboratory, Sahlgrenska University Hospital, Gothenburg, Sweden; ^12^ Hong Kong Center for Neurodegenerative Diseases, Hong Kong, Science Park, China; ^13^ Institute of Neuroscience and Physiology, University of Gothenburg, Gothenburg, Mölndal, Sweden; ^14^ Department of Neurodegenerative Disease, UCL Queen Square Institute of Neurology, University College London, London, ‐, United Kingdom; ^15^ Wisconsin Alzheimer's Disease Research Center, School of Medicine and Public Health, University of Wisconsin‐Madison, Madison, WI, USA; ^16^ UK Dementia Research Institute at UCL, London, United Kingdom; ^17^ Roche Diagnostics GmbH, Penzberg, Germany; ^18^ Roche Diagnostics International Ltd., Rotkreuz, Switzerland; ^19^ Universitat Pompeu Fabra, Barcelona, Spain; ^20^ Centro de Investigación Biomédica en Red de Bioingeniería, Biomateriales y Nanomedicina (CIBER‐BBN), Madrid, Spain; ^21^ Department of Clinical Sciences, Clinical Memory Research Unit, Lund University, Lund, Spain

## Abstract

**Background:**

Altered resting‐state functional connectivity (RSFC) has been reported in early Alzheimer's disease (AD). Graph metrics derived from RSFC networks provide valuable insights into brain organization. However, their potential in characterizing early network dysfunction and their relationship with AD biomarkers and cognitive performance remains understudied.

**Methods:**

Using RSFC data from 326 cognitively unimpaired (CU) individuals in the ALFA cohort (mean age=60.8, SD=4.74), we analyzed graph metrics in relation to cerebrospinal fluid (CSF) biomarkers. CSF Aβ42 and Aβ40 were assessed with the NeuroToolKit, a panel of exploratory robust prototype assays, while *p*‐tau181 was measured with the Elecsys® Phospho‐Tau (181P) CSF immunoassay (both Roche Diagnostics International Ltd). Interactions with age and sex were further inspected. RSFC networks were computed from 218 regions (Brainnetome atlas) using CONN, and thresholded at a density of 35%. The following graph metrics were extracted: average path length (APL), Local Efficiency (LE), Betweenness Centrality (BC), Closeness Centrality (CC), and Strength. Linear regression models assessed associations between CSF biomarkers and graph metrics, adjusting for age, sex, years of education, and *APOE*‐ε4. Interactions between CSF biomarkers and graph metrics were analyzed for their impact on longitudinal cognitive measures (Preclinical Alzheimer Cognitive Composite [PACC]; mean follow‐up=3.35 y, SD=0.53).

**Results:**

We found a positive main effect of CSF Aβ42/40 (*p* = 0.011) and *p*‐tau181 (*p* = 0.012) on BC of the Dorsal Attention Network (DAN) (Figure 1). Significant interactions between Aβ42/40 and sex were observed on the APL, CC and Strength of the Default Mode Network (DMN) (Figure 2). Finally, significant interactions were observed between Aβ42/40 and graph metrics of the DMN— APL (*p* = 0.005), LE (*p* = 0.009), and CC (*p* = 0.008)— on PACC changes (Figure 3).

**Conclusion:**

Our data suggest that, in CU individuals, soluble Ab and *p*‐tau exert opposite effects in the DAN information flow. Moreover, our interaction models suggest that a lower integration between DMN and the rest of the brain, as well as a lower centrality of DMN regions might be beneficial to preserve cognitive performance in the presence of AD. This study highlights the role of network topology in early AD and its potential to support cognitive resilience, providing potential targets for intervention.